# Aesthetic Rehabilitation of Primary Dentition with Developmental Enamel Defects Associated with Congenital Cytomegalovirus Infection and Prematurity: A 4‐Year Follow‐Up

**DOI:** 10.1111/scd.70287

**Published:** 2026-11-09

**Authors:** Luana Mota Kort Kamp, Beatriz Faria Miguel, Rafael dos Reis Moraes, Suellen Salimes Ferreira, Luciana Pomarico Ribeiro, Gloria Fernanda Barbosa de Araújo Castro

**Affiliations:** ^1^ Department of Pediatric Dentistry, School of Dentistry Universidade Federal do Rio de Janeiro Rio de Janeiro Brazil; ^2^ Centro Universitário Maurício De Nassau (UNINASSAU) Rio de Janeiro Rio de Janeiro Brazil

**Keywords:** case report, cytomegalovirus infections, dental enamel hypoplasia, premature birth

## Abstract

**Background/Aim:**

The aim of this article was to report a case of aesthetic rehabilitation of enamel hypoplasia in the primary dentition of a child born prematurely with congenital cytomegalovirus (CMV) infection, as well as its diagnosis and treatment.

**Case Presentation:**

A 3‐year‐old girl was admitted to the Pediatric Disabled Patients Clinic with an aesthetic complaint of yellow discoloration of the teeth. During the anamnesis, it was reported that the child was born prematurely (33 weeks of gestation), with low birth weight (1,310 g) and congenital cytomegalovirus infection. In the first days of life, she remained hospitalized in the intensive care unit for 68 days, requiring ventilatory support, parenteral nutrition, and prolonged antibiotic therapy. On clinical examination, the patient presented with enamel hypoplasia affecting the primary dentition, without lesions associated with dental caries. Treatment consisted of minimally invasive aesthetic rehabilitation with direct composite resin restorations on the anterior teeth and glass ionomer cement restorations on the posterior teeth. The case was followed for 4 years. During this period, the Hall Technique was performed on one of the affected primary molars.

**Conclusion:**

The diagnosis of enamel hypoplasia must include a detailed medical and dental history, together with a thorough clinical examination. Treatment selection must respect the individual characteristics of the pediatric patient. In the present case, the parents reported a perceived improvement in the child's quality of life following minimally invasive aesthetic restorative treatment.

## 1 Introduction

Developmental enamel defects (DDE) are alterations that occur during amelogenesis [1]. During this stage of dental development, hereditary, systemic, and local factors can damage ameloblasts [2]. resulting in defective and incomplete formation of the enamel matrix, known as hypoplasia [3]. The severity of the defect may vary depending on the stage and nature of dental development, as well as the timing and intensity with which the developing tooth is exposed to a specific etiological agent [4].

Congenital cytomegalovirus (CMV) infection is an uncommon yet relevant systemic risk factor for DDE. Approximately 5% of asymptomatic newborns infected with CMV present enamel hypoplasia and hypocalcification as oral manifestations [5]. This infection can be acquired during the prenatal or perinatal period and represents the most common congenital viral infection [6, 7]. The clinical manifestations of symptomatic neonates include sensorineural hearing loss, microcephaly, prematurity, and low birth weight [8].

In addition to serving as an important clinical indicator of congenital CMV infection, prematurity and low birth weight can also be considered etiological factors for DDE [2, 8, 9]. When these defects affect anterior teeth, they may compromise aesthetics [4, 10] and negatively affect the child's psychosocial well‐being, as well as increase parental dissatisfaction.

This report represents a case of long‐term aesthetic rehabilitation of enamel hypoplasia in the primary dentition, suggesting that congenital CMV infection, prematurity, and low birth weight may represent important contributing factors to DDE, such as enamel hypoplasia.

## 2 Case Report

This case report was prepared in accordance with the *CARE Guidelines* [11]. Treatment was carried out after obtaining free and informed parental consent.

### 2.1 Patient Information

A 3‐year‐old female patient with pheoderma was referred to the Pediatric Disabled Patients Clinic, Pediatric Dentistry, at the Universidade Federal do Rio de Janeiro (UFRJ), Brazil, with the chief complaint of intense yellow discoloration affecting all teeth.

During the anamnesis, the mother reported the occurrence of eclampsia during pregnancy and a vaginal delivery. The patient was born prematurely at 33 weeks of gestation, with low birth weight (1,310 g). After birth, the patient was hospitalized for 68 days in a neonatal intensive care unit (NICU), including 45 days in an incubator with ventilatory support and 21 days receiving total parenteral nutrition (TPN). The patient presented with neonatal sepsis and cholestasis and was diagnosed with congenital CMV infection, requiring prolonged antibiotic therapy. There was no family history of systemic disease or dental alterations. At present, the child has precocious thelarche and regularly uses vitamin supplements. She continues to be periodically monitored by a specialized medical team. At the initial consultation, both the patient and her mother expressed significant dissatisfaction with the tooth discoloration.

### 2.2 Clinical Findings

Clinical examination revealed that the child presented with gait abnormalities, cognitive impairment, and mild hearing loss. Intraoral examination revealed complete primary dentition, absence of dental caries, and normal soft tissues (Figure 1). All primary teeth showed reduced enamel thickness, consistent with hypoplasia [12]. The extent of the affected tooth surface varied among tooth groups: the incisors showed involvement of at least two‐thirds of the surface, whereas the canines showed involvement over less than one‐third and molars showed involvement of at least one‐third but less than two‐thirds of the surface [13]. The patient did not report pain or dentin hypersensitivity.

**FIGURE 1:**
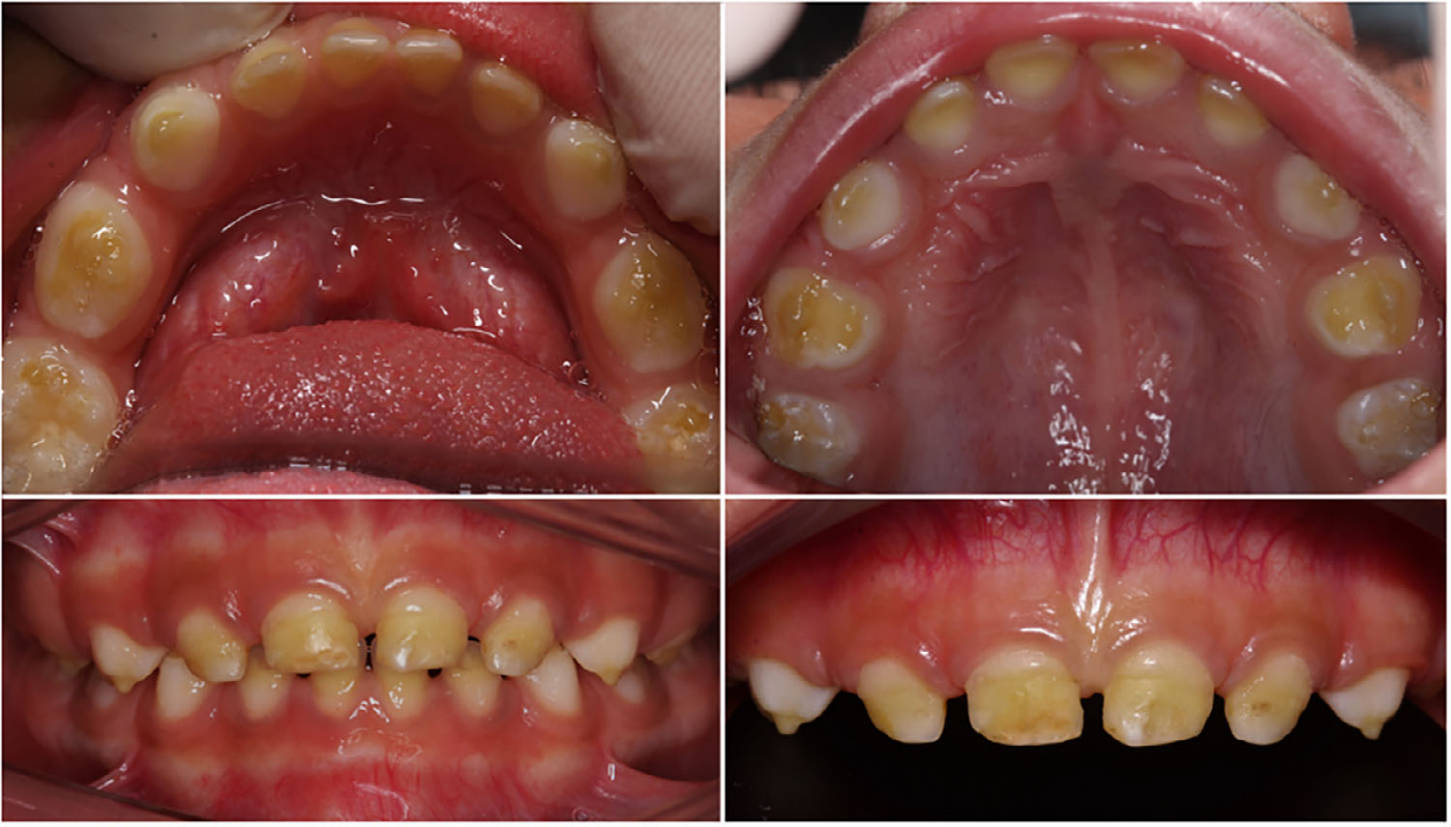
Initial clinical condition of the patient.

### 2.3 Diagnostic Reasoning

The diagnostic hypotheses considered for this case were amelogenesis imperfecta, enamel hypoplasia, and early childhood caries. After thorough clinical examination and exclusion of differential diagnoses, the patient was diagnosed with enamel hypoplasia. No radiographic examinations were required to complement the diagnostic process.

### 2.4 Therapeutic Intervention

During dental care, the patient exhibited definitely negative behavior (–) [14]. Therefore, a minimally invasive approach was adopted, performed by the dentist with assistance under active protective stabilization provided by the mother [15]. Direct restorations were performed under relative isolation on the maxillary (53, 52, 51, 61, 62, 63) and mandibular (73, 72, 71, 81, 82, 83) teeth. The teeth were cleaned by prophylaxis with pumice and water, conditioned with phosphoric acid for 15 s (Condak 37%, FGM, Santa Catarina, Brazil), rinsed for 30 s, and dried with absorbent paper. Conventional adhesive procedures were then performed (Ambar, FGM, Joinville, Santa Catarina, Brazil), followed by direct composite resin restorations (FiltekTM Z350 XT, 3 M ESPE, Saint Paul, MN, USA), finishing, and polishing.

In addition to the aesthetic restorations of the anterior teeth, the hypoplastic areas of the maxillary (55, 54, 64, 65) and mandibular (75, 74, 84, 85) posterior teeth were sealed using a high‐viscosity conventional glass ionomer cement. The teeth were cleaned by prophylaxis with pumice and water, conditioned with polyacrylic acid (Ketac Molar Easy Mix A3, 3 M ESPE, Saint Paul, MN, USA) for 10 s, rinsed for 20 s, and dried with absorbent paper. The hypoplastic areas were then sealed with conventional glass ionomer cement (Ketac Molar Easy Mix A3, 3 M ESPE, Saint Paul, MN, USA). Petroleum jelly was applied to the restorations for surface protection, followed by occlusal registration with articulating paper and removal of excess material using hand instruments. The procedures were performed by sextants over 6 clinical visits to complete the proposed treatment (Figure 2), achieving satisfactory aesthetic and functional outcomes.

**FIGURE 2:**
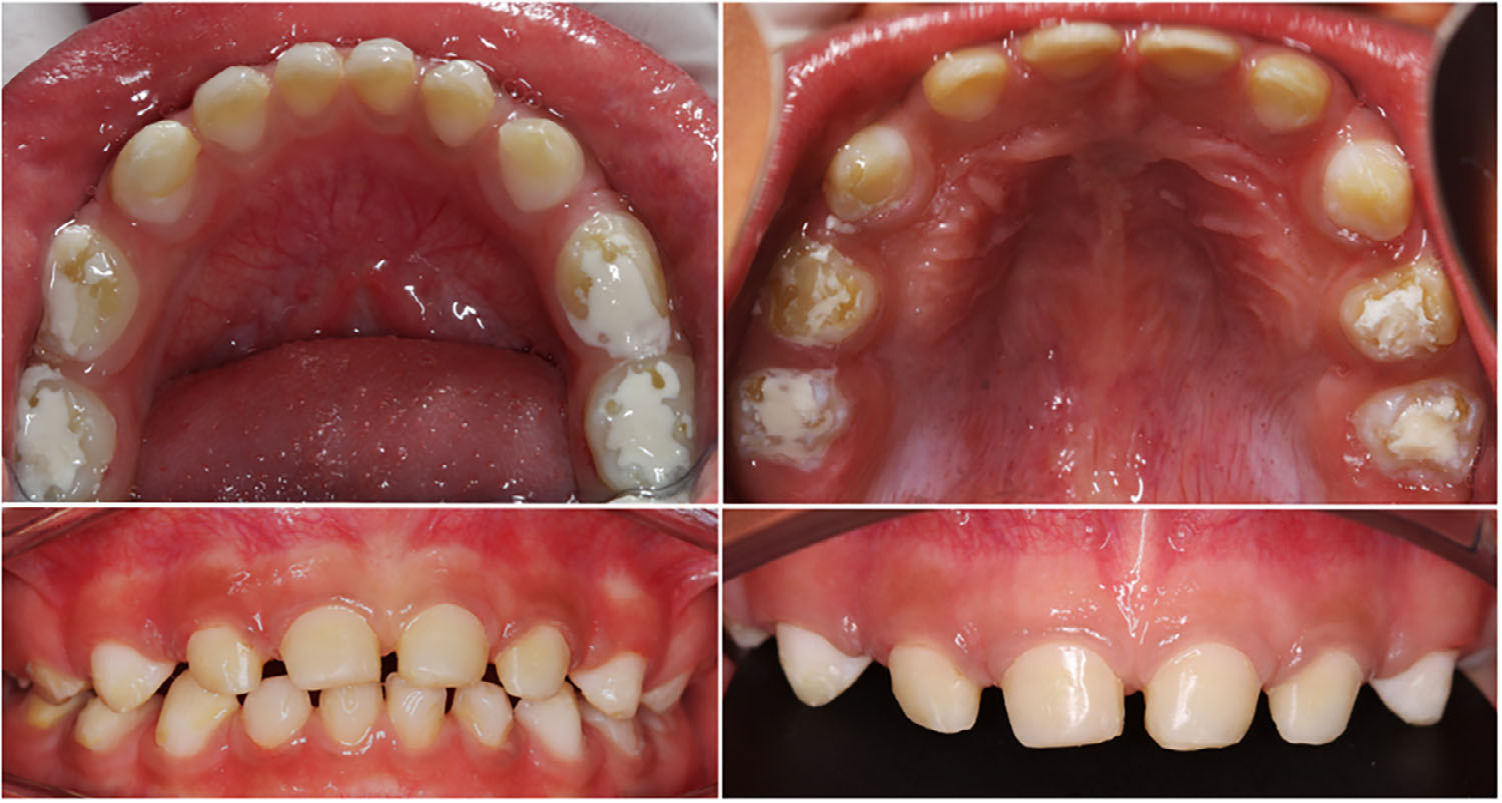
Final clinical appearance of the restorations.

### 2.5 Follow‐Up

The patient was followed for 4 years after aesthetic and functional rehabilitation, until the eruption of the permanent incisors and molars. During this period, greater loss of marginal integrity was observed in a maxillary second primary molar. Therefore, the Hall Technique was indicated to provide greater coverage and protection of the remaining tooth structure (Figure 3). At the final consultation, the erupted permanent teeth also exhibited DDE, which may be explained by a systemic condition occurring during the gestational period. The patient received oral health instructions and was advised to attend regular follow‐up appointments.

**FIGURE 3:**
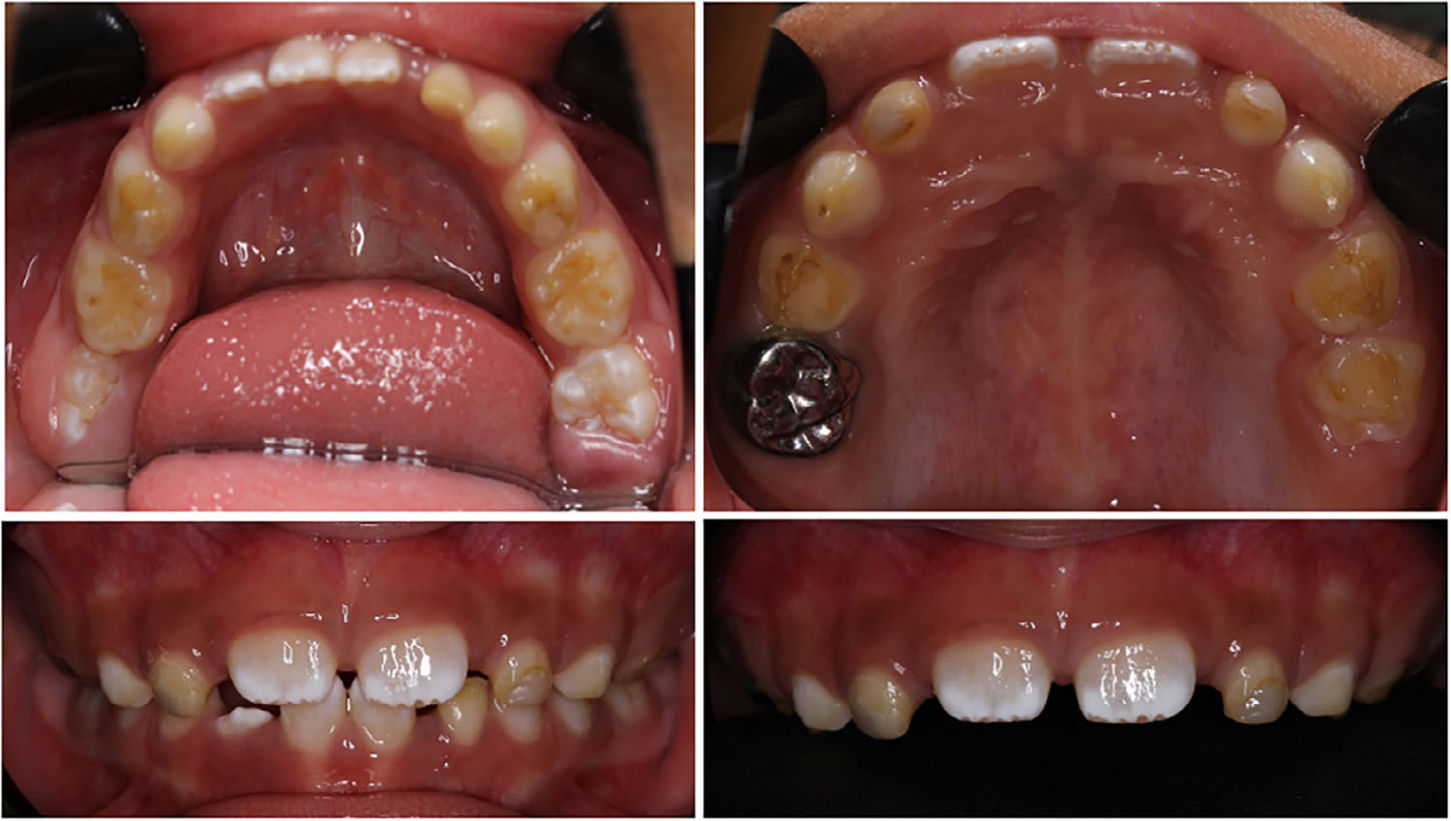
Clinical condition after 4 years of follow‐up, showing mixed dentition.

## 3 Discussion

This report emphasizes the importance of considering prenatal, perinatal, and postnatal history, because events occurring during these periods may aid in the diagnosis of DDE. It also highlights the relevance of long‐term follow‐up and aesthetic management of the primary dentition in rare cases associated with congenital CMV infection and prematurity. The development of enamel hypoplasia in primary teeth may be associated with a range of adverse events in early life that occur during the neonatal and postnatal periods, such as premature birth, low birth weight, nutritional disorders, systemic conditions, local trauma, and medication use [16].

Congenital CMV infection was first described as a possible etiological factor for the development of DDE in 1982. No reports in the literature have demonstrated this association to date [17]. In an experimental study in rats, Jaskoll et al. (2010) demonstrated that CMV induces enamel defects and that defect severity is directly associated with the stage of tooth formation and duration of the infectious process. In other words, the earlier the stage of tooth development is and the longer the duration of the infection, the greater the severity of the defect.

In the present case, the child was born with congenital CMV infection, and infection occurred during the second trimester of pregnancy. These findings suggest that the development of hypoplasia affecting all primary teeth is related to the infection. Some studies also report that infants with asymptomatic congenital CMV infection present less severe and extensive defects. The fact that the patient in this report was symptomatic may explain the greater extent and severity of dysplastic areas affecting all teeth [18].

Prematurity and low birth weight, in addition to being important manifestations of congenital CMV infection, are also considered potential etiological factors in the development of DDE in primary teeth [19]. Since the patient in the present report was born prematurely, with low birth weight, and diagnosed with congenital CMV infection, the combination of prenatal and postnatal factors may have affected tooth formation and mineralization, favoring the development of quantitative defects in all teeth.

Most premature infants with low birth weight require care in NICUs, including antibiotic therapy, ventilatory support, TPN, and vitamin supplements [20]. These factors may be associated with an increased risk of DDE. Therefore, their contribution cannot be excluded in the present case, considering that the patient was hospitalized in the NICU for 68 days, required ventilatory support and TPN, and received antibiotics and vitamin supplements. Cortines et al. (2018) also reported that newborns admitted to the NICU presented a higher prevalence of enamel hypoplasia, and that factors such as duration of hospitalization, antibiotic use in the NICU, and need for intubation increased the number of teeth affected by DDE.

Differential diagnoses must be considered when enamel alterations affect all teeth. Amelogenesis imperfecta is a rare genetic condition that affects enamel structure, quantity, and composition in both primary and permanent dentition [21]. Since no family history of this condition was reported in the present case, this diagnostic hypothesis was excluded. Early childhood caries is defined as the presence of one or more decayed, missing, or restored surfaces in any primary tooth of a child younger than 6 years [22]. This hypothesis was also excluded in the present report, since the lesions were primarily located in areas not prone to dental biofilm accumulation, were not associated with active white spot lesions, and did not present demineralized tissue characteristic of active caries lesions.

Although the patient in the present case did not present dental caries, some studies report that hypoplastic areas may be highly susceptible to caries [23]. The absence of lesions may be related to the child's young age and low consumption of sugary foods. However, a preventive approach is required to promote oral health in both public and private healthcare settings. Dentists must be aware that children with DDE may require more frequent dental visits and periodic clinical follow‐up to reduce the risk of caries development [24].

The treatment of hypoplastic and hypomineralized teeth remains a challenge. Current approaches aim to prevent tooth structure loss, provide aesthetic and functional rehabilitation, and control hypersensitivity [25]. Early diagnosis, combined with assessment of disease severity, patient age, aesthetic concerns, psychological factors, and expectations of the patient and caregivers, is essential for developing an individualized treatment plan [26]. In the present case, a minimally invasive approach was selected using direct restorations without prior tooth preparation. Composite resin was used in the anterior region due to aesthetic concerns, while posterior teeth were treated with glass ionomer cement to prevent caries development. This approach enabled rapid and conservative treatment in a patient with behavioral challenges, minimizing the need for sedation or general anesthesia, which might be required with more invasive procedures.

A limitation of this case report is the lack of radiographic documentation. At the initial assessment, no clinical findings indicated an immediate need for radiographic examination, and the child's limited cooperation also posed an additional challenge to obtaining adequate radiographic images. During follow‐up, a panoramic radiograph was requested. However, the patient did not return to the service. Therefore, although the diagnosis and follow‐up of the DDE were primarily based on clinical findings, radiographic documentation could have complemented the clinical assessment and provided a more comprehensive longitudinal record of the case.

The aesthetic impact of DDE may negatively affect children's quality of life, interfering with socialization, school performance, self‐esteem, and even contributing to bullying [27]. Therefore, it is essential to obtain detailed information during the anamnesis to identify psychosocial factors and assess the child's and family's perception of the condition [28]. Understanding these aspects is essential for accurate diagnosis and for establishing a treatment plan tailored to each patient's individual needs and concerns.

## 4 Conclusion

The diagnosis of enamel hypoplasia and other DDE must include a detailed medical and dental history, a thorough clinical examination, and all potential etiological factors associated with this condition. Treatment selection must consider the individual characteristics of the pediatric patient, including age, behavior, psychosocial factors, aesthetic impact, risk of bullying, and parental perspective. In the present case, the parents reported a perceived improvement in the child's quality of life following minimally invasive aesthetic restorative treatment. Furthermore, continued follow‐up is essential, given the risk of DDE affecting the permanent dentition.

## Author Contributions

Luana Kort‐Kamp: Conceptualization, Methodology, Investigation, Writing – original draft, and Patient Management. Beatriz F Miguel and Suellen S Ferreira: Author 2: Investigation, Patient Management, Data Curation, and Writing – review & Editing. Rafael R Moarae and Luciana P Ribeiro: Methodology, Visualization, and Writing – review & Editing. Gloria F B A Castro: Conceptualization, Supervision, Validation, and Writing – review & Editing. All authors read and approved the final version of the manuscript.

## Conflicts of Interest

The authors declare no conflicts of interest related to this work.

## Supporting information




**Supporting Information**: scd70287‐sup‐0001‐SuppMat.docx
