# Supplementary material for: Aesthetic Rehabilitation of Primary Dentition with Developmental Enamel Defects Associated with Congenital Cytomegalovirus Infection and Prematurity: A 4‐Year Follow‐Up
Source: Spec Care Dentist. 2026 Nov 9;46(6):e70287. doi: 10.1111/scd.70287 (PMC13747198; doi:10.1111/scd.70287)
Supplement: Supplementary file 1 [file SCD-46-0-s001.docx]

**List of Figures**

**Figure 1:** Initial clinical condition of the patient.

**Figure 2:** Final clinical appearance of the restorations.

**Figure 3:** Clinical condition after 4 years of follow-up, showing mixed dentition.
